# Pinpointing genes underlying annual/perennial transitions with comparative genomics

**DOI:** 10.1186/s12864-016-3274-1

**Published:** 2016-11-15

**Authors:** Andrew J. Heidel, Christiane Kiefer, George Coupland, Laura E. Rose

**Affiliations:** 1 Institute of Population Genetics, Heinrich-Heine-Universität, Universitätsstraße 1, Düsseldorf, D-40225 Germany; 2 Faculty of Biology & Pharmacy, Department of Bioinformatics, University of Jena, Ernst Abbe Pl 2, Jena, D-07743 Germany; 3 Cluster of Excellence on Plant Science, Düsseldorf, 40225 Germany; 4 Department of Plant Developmental Biology, Max Planck Institute for Plant Breeding Research, Carl-von-Linné Weg 10, Cologne, D-50829 Germany

**Keywords:** Gene loss, Life history, Plants, Evolution

## Abstract

**Background:**

Transitions between perennial and an annual life history occur often in plant lineages, but the genes that control whether a plant is an annual or perennial are largely unknown. To identify genes that confer differences between annuals and perennials we compared the gene content of four pairs of sister lineages (*Arabidopsis thaliana*/*Arabidopsis lyrata*, *Arabis montbretiana*/*Arabis alpina*, *Arabis verna*/*Aubrieta parviflora* and *Draba nemorosa*/*Draba hispanica*) in the *Brassicaceae* in which each pair contains one annual and one perennial, plus one extra annual species (*Capsella rubella*).

**Results:**

After sorting all genes in all nine species into gene families, we identified five families in which well-annotated genes are present in the perennials *A. lyrata* and *A. alpina*, but are not present in any of the annual species. For the eleven genes in perennials in these families, an orthologous pseudogene or otherwise highly diverged gene was found in the syntenic region of the annual species in six cases. The five candidate families identified encode: a kinase, an oxidoreductase, a lactoylglutathione lyase, a F-box protein and a zinc finger protein. By comparing the active gene in the perennial to the pseudogene or heavily altered gene in the annual, dN and dS were calculated. The low dN/dS values in one kinase suggest that it became pseudogenized more recently, while the other kinase, F-box, oxidoreductase and zinc-finger became pseudogenized closer to the divergence between the annual-perennial pair.

**Conclusions:**

We identified five gene families that may be involved in the life history switch from perennial to annual. Considering the dN and dS data and whether syntenic pseudogenes were found and the potential functions of the genes, the F-box family is considered the most promising candidate for future functional studies to determine if it affects life history.

**Electronic supplementary material:**

The online version of this article (doi:10.1186/s12864-016-3274-1) contains supplementary material, which is available to authorized users.

## Background

Annuals have evolved many times from perennial ancestors [39] and the reverse has also occurred probably to a more limited extent [3]. These switches between life histories have produced closely related species where one is an annual and one a perennial [10, 25]. Annualism can evolve as an adaptation to xeric environments [10, 25], and loci contributing to the switch can be identified, although their number is variable [10, 14, 26, 28].

Differential behavior of meristems, whereby some stay vegetative while others transition to flowering, is one important aspect that distinguishes perennial from annual plants [1]. Genes have been found that inhibit flowering in some meristems [21, 29, 43]. In perennial Brassicaceae species this behavior is conferred by orthologues of the *A. thaliana* gene *FLOWERING LOCUS C*, which inhibit flowering until the plant is exposed to an extended period of cold that mimics winter, a process referred to as vernalization [20, 43]. However less is known about the genetics of other differences between annuals and perennials, such as senescence patterns.

A common mechanism of evolutionary change is chromosomal duplication and gene loss [4, 27, 37, 40]. These gains and losses have shown the potential to influence morphology [8], and gains and losses include genes affecting flower morphology [23]. Losses of the same gene at different positions in a phylogeny can explain repeated occurrence of the same trait, such as differences in leaf morphology [35, 42]. Since this mechanism could also affect life history changes we have investigated whether it is involved in the switch to annualism. Although other evolutionary mechanisms such as expression changes and positive selection may also be important, given the known loss in genes between the perennial *Arabidopsis lyrata* and the annual *Arabidopsis thaliana* [15, 32], we concentrate on gene loss as a starting point. By gene loss we mean changes that remove the function of a gene from the genome. This can include deletion of a gene from the genome, pseudogenization of a gene or changes to a gene that are so substantial, such as removal or replacement of complete protein domains, that they likely drastically change the function. Furthermore, we restrict our study to species in the Brassicaceae*,* in which the transition from annuality to perenniality occurred multiple times independently [18].

As a basis for comparisons we use four annual/perennial sister species plus one additional annual functioning as an outgroup. The four pairs are: (annual/perennial) 1) *A. thaliana*/*A. lyrata*, 2) *Arabis montbretiana*/*Arabis alpina*, 3) *Draba hispanica*/*Draba nemorosa* 4) *Arabis verna*/*Aubrieta parviflora*. By finding genes that have been lost in annuals in all four species pairs we aim to find candidate genes that contribute to the perennial life history but are not required by annuals.

## Methods

### Plant material

Seeds of perennial or annual taxa additional to *A. thaliana*, *A. lyrata*, *A. alpina* or *A. montbretiana* were obtained from Marcus Koch, COS, Heidelberg University/Germany (Additional file 1: Table S1). Seeds of *A. montbretiana* were obtained from Birol Mutlu, Inonu University, Turkey. Plants were grown under greenhouse conditions (constant temperature of 20 °C, 16h light) on standard soil (Balster Einheitserde) with a slow release fertilizer (osmocote).

### Phylogenetic tree construction

To construct the phylogenetic tree, two loci were used: ITS and trnLF (accession numbers are given in Additional file 2: Table S2). Individual loci were aligned with MUSCLE v3.8.31 [7] then inspected by eye and nonalignable sections were deleted. The alignment is available from the Dryad Digital Repository: http://dx.doi.org/10.5061/dryad.5pv1k. The two loci were concatenated and the maximum likelihood tree was constructed in MEGA6 using the HSK85 model [11, 41]. Uniform rates were used among sites, all sites were used and the branch swap filter was “very strong.” Branches subtending each annual/perennial species pair were supported with bootstrap values greater 95% based on 2000 bootstrap replicates.

### Assembly and collection of gene sets

Coding regions and protein sequences of *C. rubella* [36], *A. thaliana* [19] and *A. lyrata* [15] were downloaded from phytozome.net (PhytozomeV9). The *A. montbretiana* coding sequences were determined using Augustus 2.7 [38] which uses a genetic assembly (*A. montbretiana* contig assembly genebank accession LNCH00000000) combined with RNAseq data (RNAseq data obtained from rosette leaves, cauline leaves, cotyledons, seedlings, floral buds, vegetative apices) from unpublished data. For the other representatives of annual/perennial sister groups (*D. hispanica*, *D. nemorosa*, *A. parviflora and A. verna*) RNA was extracted using the RNeasy Mini Kit (Qiagen) from leaf tissue and DNase treated (Ambion) according to the manufacturer’s manuals. Library construction and sequencing was performed by the Max Planck Genome Center (Cologne).

The reads from four species (*D. hispanica*, *D. nemorosa*, *A. parviflora*, *A. verna*) were assembled by Trinity (Release r20131110) which performs *de novo* assembly and annotation of genes from RNAseq reads [9]. In Trinity all default settings were used. Open reading frames were obtained from Trinity output using the Trinity sub-program transdecoder with default values. The longest version of each transcript was taken as the primary transcript. Sequences from an earlier unpublished version of the *A. alpina* genome were used to begin this study; later the genome was published [44].

### Sorting into gene families

OrthoMCL version 1.4 was used with default values to sort all protein coding genes into families [24]. After the determination of the families with genes missing from annuals, some families were removed based on misannotation. This misannotation was determined based on one of the following observations: 1) a complete gene was found with high identity in the annual sister species, 2) annotation in perennial was in the wrong frame.

Families were further eliminated from consideration as a measure to remove potential false positives where the gene in the annuals with only transcriptome data was incomplete. In this case the gene in the annual would be not be grouped with the family by OrthoMCL due to the missing sequence. The phylogenetic tree illustrates that the species with only transcriptome data are closer to *A. alpina* than to *A. lyrata.* Therefore, we removed those families where the *A. alpina* BLAST hit against the annuals with transcriptome data was much better than the BLAST hit of the *A. alpina* gene to the *A. lyrata* gene. Specifically if the blastn or blastp *E*-value between a sequence from *A. alpina (perennial)* to the annuals for which only transcriptome data was available was one order of magnitude lower than the BLAST hit between the *A. lyrata* gene and *A. alpina* gene, the family was eliminated. For these BLAST analyses, the default parameters were used in BLAST version 2.2.28+. All further uses of BLAST (other than when NCBI BLAST is specifically mentioned) use this version. NCBI BLAST queries were performed to determine if genes were transposons, self-incompatibility or ribosomal genes using default values and using all available sequences as a database.

To determine syntenic regions within the pairs *A. thaliana*/*A. lyrata* and *A. montbretiana*/*A. alpina*, bidirectional best hits were determined. This was done with blastn with default parameters using one genome’s set of genes as the blastn database and querying it with all the genes from the other in the pair. Then the species in each pair were switched and the blastn was performed again. Two genes were determined to be bidirectional best hits if each gene was the best blastn hit of the other. Searches for pseudogenes within the syntenic regions or in the entire genome were performed with blastn and default parameters using the syntenic region and the whole genome respectively as blastn databases.

The final set of gene families were annotated with blast2go version 3.0.8 using default values [5]. To calculate dN (nonsynonymous substitutions per nonsynonymous site) and dS (synonymous substitutions per synonymous site) values in the four candidate families with pseudogenes, the pseudogenes in the annuals were compared to the complete genes in the perennials with the following method: 1) The part of the pseudogene that was alignable to the real gene was aligned as a protein sequence with MUSCLE and then converted back to a nucleotide sequence 2) the alignment was adjusted by eye 3) dN and dS were calculated by PAML Perl module yn00 [46]. For the calculation of the dN and dS values for all 10,533 families with *A. thaliana*, *A. lyrata*, *A. montbretiana* and *A. alpina*, custom Perl scripts aligned the genes with MUSCLE and then calculated dN and dS with PAML Perl module yn00. MUSCLE alignments were performed on protein sequences and then converted back to nucleotide sequences. Values were not calculated for 20 families due to poor alignments, and the dN/dS ratio was not calculated from an additional 100 families due to a dS value of 0.

## Results and discussion

### Phylogenetic positions of species

**Fig. 1:**
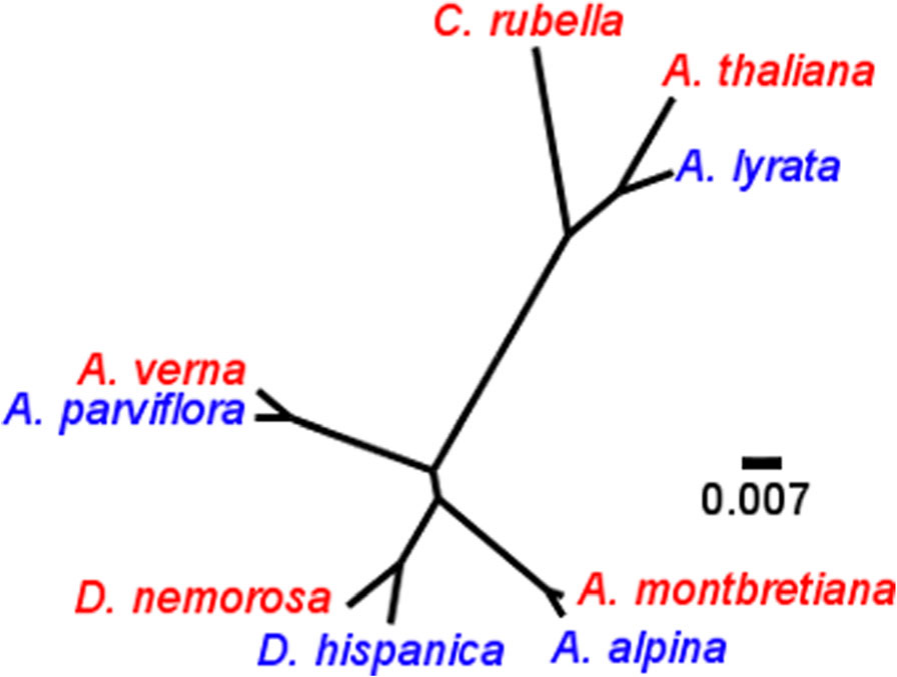
Phylogenetic tree of the nine species based on two genes: ITS and trnLF. Annual species are indicated in red and perennials in blue. The tree was inferred using Maximum Likelihood and the scale bar indicates substitutions per site

The phylogentic positions of all species used in this analysis were already known [13, 17, 18], but a phylogenetic tree was constructed to specifically illustrate the phylogenetic relationships of the nine species and the four species pairs differing in life history. The phylogenetic tree is based on the concatenated sequences of two loci using a maximum likelihood method (Fig. 1) and is in agreement with previously published phylogenies referenced above. The tree shows that the four pairs of species, each containing one annual and one perennial, are all more closely related to each other than to any of the other species. Therefore this set of species is an excellent group to investigate the transition between perennials and annuals.

### Grouping of species into families and filtering

**Fig. 2:**
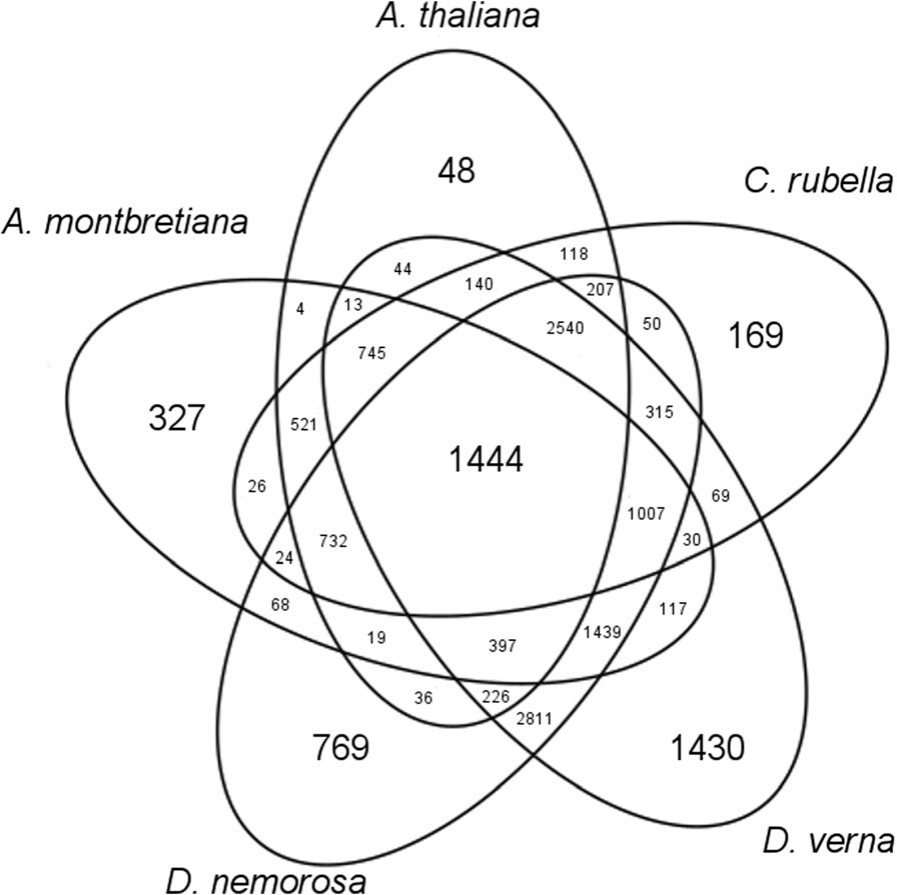
Venn diagram of gene families in which at least one or more annual species is missing. Each oval corresponds to an annual species and states the number of gene families that do not contain a gene from that species (*e.g*. there are 48 families that are missing *A. thaliana* genes only and 118 families that are missing *A. thaliana* and *C. rubella* genes)

**Fig. 3:**
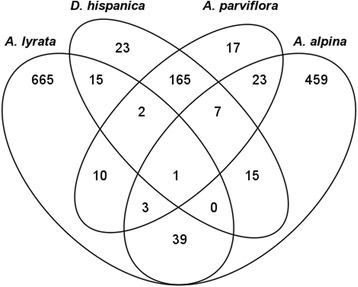
Venn diagram of gene families in which all annual species are missing, but present in at least one or more perennial species. Starting from the 1444 gene families in which all annual species are not present, each oval corresponds to a perennial species and states the number of gene families that contain that species (*e.g.* there are 665 families that contain only *A. lyrata*, and 15 that contain *A. lyrata* and *D. hispanica*)

To detect genes that have been lost or pseudogenized in annuals, the protein sequences from the primary transcripts from nine species were first grouped into gene families by OrthoMCL. It is noted that the gene families generated here and referred to hereafter as gene families are generally smaller and do not necessarily correspond to other descriptions of gene families in the literature. From these gene families, there are 1444 gene families that contain at least one gene from a perennial species but contain no genes from annual species. The distribution of gene families containing no members from the annual species is visualized in Fig. 2. The gene families present in at least one perennial but missing in all annual species are further investigated in Fig. 3, in which the distribution of genes in the perennial species is illustrated. Families of interest for the transition from perennial to annual should be present in perennials and absent in annuals. A single family has at least one gene from all perennials and has no genes from annual species (the intersection of all four perennial species in Fig. 3). Since only transcriptome data (and not full genome information) is available for two perennial species (*D. hispanica* and *A. parviflora*), it is useful to also consider those gene families present in the other perennials, but that were not detected in *D. hispanica* or *A. parviflora*. This relaxed assumption includes an additional 42 families. These families when combined with the single family present in all perennials, gives a total of 43 families that are further investigated.

Differences are apparent in the distribution of gene families between species (Figs. 2 and 3). For families in which the gene is missing in one annual species only, the largest counts are for genes missing in *D. nemorosa* and A*. verna,* while the fewest are observed in *A. thaliana* (Fig. 2). This variation likely reflects the difference in the quality of the genomic information; lowest quality for those for which only transcriptome data exist and highest quality for the model plant *A. thaliana*. The largest count of gene families missing from two annual species is for *D. nemorosa* and A*. verna* (Fig. 2). For families present in only one perennial, the largest values are in *A. lyrata* and *A. alpina* (Fig. 3).

While these differences could result from real genomic differences, it is more likely that the quality of the genome and transcriptome assemblies is the reason. *A. thaliana* has a better quality genome than *A. alpina*, *A. montbretiana* and *A. lyrata,* and all three genomes have better annotated gene contents than the two *Draba* species for which only RNAseq data are available. One indication of this quality is that all *A. thaliana* sequence is placed on the five nuclear chromosomes or the mitochondrial and chloroplast genomes while *A. montbretiana* sequence is distributed on 28,936 contigs, and the most recent version of *A. lyrata* (V10.3) is on 3648 scaffolds. Furthermore, *A. thaliana* is the least likely to have misannotated genes whereas *D. nemorosa* and A*. verna* are more likely to have missing genes.

The functions of the 43 families, absent in annuals and having at least one gene in both *A. lyrata* and *A. alpina,* were determined based on annotation of the *A. lyrata* and *A. alpina* genomes, BLAST searches at NCBI and InterProScan. Based on this annotation, families that included transposons, self-incompatibility and ribosomal genes were removed since these families are not likely to underlie transitions between perennials and annuals. Furthermore the genes were inspected by eye and gene families including misannotated genes were eliminated (see Methods for criteria). *A. lyrata* genes were further checked against the newest annotation and genes not annotated in the newest version were eliminated. *A. alpina* genes were checked against the published version in GenBank and those not present were eliminated. It was further determined whether incomplete genes might be present in the RNAseq data from *D. nemorosa* or A*. verna* that if complete would have been included in the family. Since these two species are closer to *A. alpina* than to *A. lyrata,* if the *E*-value of the BLAST hit between the *A. alpina* gene and the best BLAST hit in the entire gene set of *D. nemorosa* or A*. verna* was one order of magnitude smaller than the *E*-value between the *A. alpina* gene and the *A. lyrata* gene, the family was eliminated. After removal of families based on all of the above criteria, five families remained, all of which contain genes only in *A. alpina* and *A. lyrata* and are missing from all annuals.

### Identification of pseudogenes

Information on synteny was used to understand the fate of the missing genes in the annual species, *A. thaliana* and *A. montbretiana*. Synteny could not be investigated in the other species pairs since only transcriptome data was available. If the syntenic location of the missing gene could be found, it could be determined if the gene has been pseudogenized. Starting with the genes from the remaining families in *A. alpina* and *A. lyrata*, the adjacent gene on each side was found. Then the bidirectional best hits (BBH) in the sister species to the adjacent genes were found using BLAST. If BBHs exist for both adjacent adjacent genes then the sequence between the genes was scanned for genes with homology to the candidate perennial specific genes in *A. lyrata* and *A. alpina*. If only one BBH exists then the next four genes in the correct direction from the BBH were checked for homology. If no gene had homology then the sequence between the two BBHs or 40 kb on the correct side of the single BBH was searched by blastn to find sequence homologous to the genes in *A. lyrata* and *A. alpina*. If no homologous region was found in these areas, the entire genome was searched by blastn. In four of the five families, a pseudogene could be found in at least one of the species (Table 1). In the cases where no pseudogene could be found, the gene is either largely deleted from the genome to the extent that no homology could be found or the region containing the gene was not sequenced.

**Table 1:** Top five candidate genes for perennial to annual transitions via gene loss

Family	Function	*A. lyrata*/*A. alpina* genes ^a^	real gene in *A. thaliana*/*A. montbretiana* ^*b*^	pseudogene in syntenic region ^c^	pseudogene in nonsyntenic region ^d^
1	Kinase	1/2	0/0	1/2	-/-
2	Oxidoreductase	1/1	0/0	1/0	-/0
3	Lactoylglutathione Lyase	1/1	0/0	0/0	0/0
4	F-box protein	1/1	1/0	-/1	-/-
5	Zinc finger	1/1	0/0	0/0	1/0

^a^ Number of genes in the perennial species *A. lyrata* and *A. alpina* in the gene family

^b^ Number of real genes (not pseudogenes) found in the syntenic region of the annual species, *A. thaliana* or *A. montbretiana,* compared to their perennial sister species, *A. lyrata* and *A. alpina,* respectively

^c^ Number of pseudogenes found in syntenic regions of *A. thaliana* or *A. montbretiana*

^d^ Number of pseudogenes found in nonsyntenic regions

### Gene annotation

The five families were annotated with blast2go and the proposed functions are given in Table 1. Although a general function could be assigned to all families using blast2go a more exact function is difficult to establish without functional studies, and there have been no functional studies of these genes or of closely related genes. The accession numbers of all genes in these families are given in Additional file 3: Table S3. The first family contains oxidoreductases that are involved in metabolism and defense [2], and their enzymatic activity can regulate other enzymes and transcription factors [33]. The lactoylglutathione lyase glyoxalase I-like family is involved in tolerance to salinity and heavy metals [30].

Two families are likely involved in signal transduction as one encodes kinases and the other F-box proteins. The closest *A. thaliana* blastp hit (AT3G57770) to the kinase family also has been annotated as a kinase, contains kinase IPR domains and is expressed in stems, guard cells and the shoot system [31, 34]. Although blast2go annotates the two *A. alpina* genes and the *A. lyrata* gene as a kinase none of these three genes contain a known IPR kinase domain, so the functional determination is not certain. In the F-box family, both the *A. lyrata* and *A. alpina* gene contain the F-box domain (IPR001810), the F-box associated domain (IPR006527) and the F-box associated interaction domain (IPR017451). The *A. lyrata* gene additionally contains a galactose oxidase domain which is common in F-box genes [6]. There is a homologous gene in a syntenic region in *A. thaliana* (AT3G16020) which is not the best BLAST hit due to the *A. lyrata* gene being 366 aa and the *A. thaliana* gene being 98aa. Although the gene is not pseudogenized in *A. thaliana,* its function is likely altered from the loss of over 2/3rds of its length compared to *A. lyrata* including the loss of the F-box domain, although it retains the F-box associated domain. To our knowledge there have been no experimental functional studies of the *A. thaliana* gene, but based on bioinformatic analysis the *A. thaliana* gene is involved in regulation of chromosome organization and vernalization response [12]. The *A. thaliana* gene also includes a candidate Phospholipase C domain (IPR000909) which is not found in the *A. lyrata* and *A. alpina* genes, but has been found in at least one other F-box gene (AT1G30925).

One family includes potential transcription factors as they encode zinc fingers. The *A. thaliana* best blastp hit (AT1G60500) contains a number of Dynamin related domains (IPR003130, IPR001401, IPR022812 and IPR000375), however these domains are all missing in the *A. lyrata* and *A. alpina* genes.

### Dating of pseudogenization

**Fig. 4:**
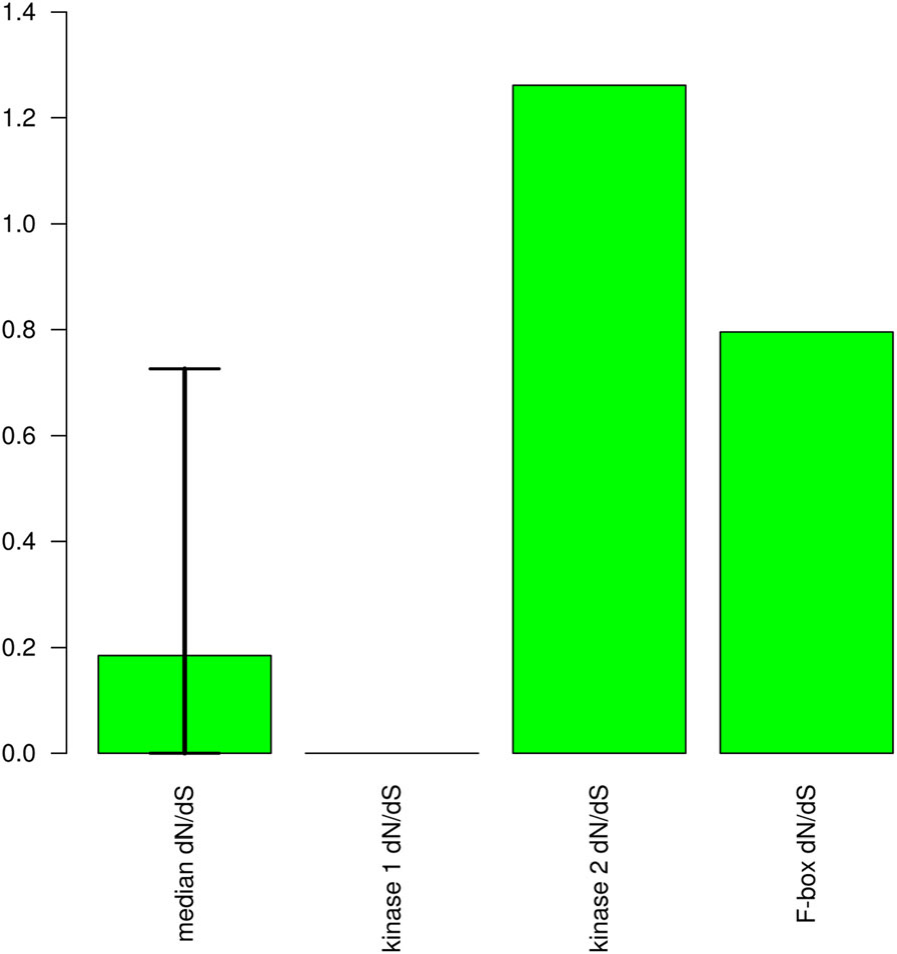
dN/dS ratios for the species pair: *A. montbretiana* and *A. alpina*. The first column is the median for a comparable group of gene families with 5th to 95th percentiles indicated above and below the median. The remaining columns give the dN/dS ratios from single pseudogenes in *A. montbretiana* versus coding genes in *A. alpina*. Kinase 1 refers to AALP_AA5G219100 and kinase 2 refers to AALP_AA1G091400

**Fig. 5:**
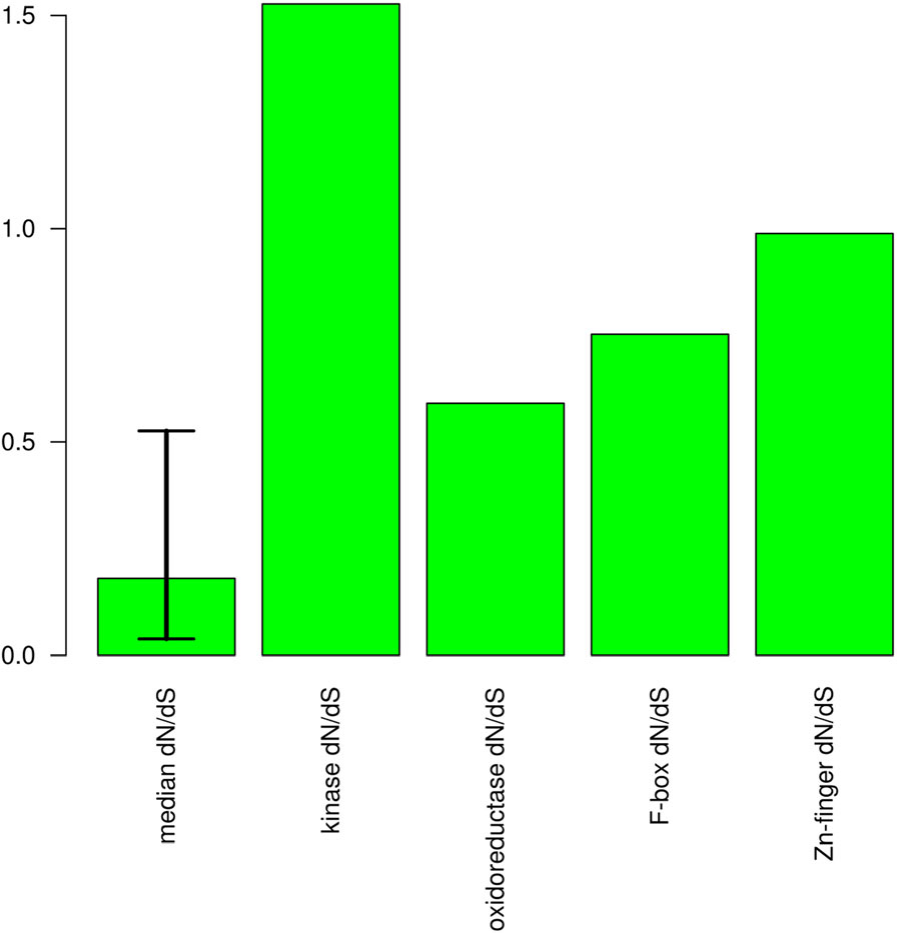
dN/dS ratios for the species pair: *A. thaliana* and *A. lyrata*. The first column is the median for a comparable group of gene families with 5th to 95th percentiles indicated above and below the median. The remaining columns give the dN/dS ratios from single pseudogenes in *A. thaliana* (or a coding gene in the case of the F-box gene) versus coding genes in *A. lyrata*

To estimate the date of the pseudogenization of the genes, the dN/dS ratios between the genes in perennials and the pseudogenes (and the one real gene) were calculated (Figs. 4 and 5). In both figures a single dN/dS ratio is calculated from the pseudogene in the annual versus the coding gene in the perennial. Although the pseudogenes are no longer coding regions, they could be aligned to the real genes in the perennials and any positions with stop codons in the pseudogene were deleted. For the large majority of genes in the genome the dN value is lower than the dS value due to purifying selection leading to a dN/dS ratio less than one. The dN values between the genes and pseudogenes are expected to be higher than between orthologous functional genes, due to the relaxation of purifying selection on nonsynonymous sites in the pseudogenes. However, purifying selection was likely acting until the pseudogenization occurred and is still acting on the perennial gene, so purifying selection on dN is not completely relaxed. If the pseudogenization happened recently, dN may still be much lower than dS. Conversely if the pseudogenization is older, the dN may be closer to or even higher than dS leading to a dN/dS ratio close to or greater than one. For comparison, a set of families was selected that was similar in composition to the families with pseudogenes. This set of families consists of all 10,533 families that at a minimum contain single genes from *A. thaliana*, *A. lyrata*, *A. montbretiana* and *A. alpina*. The 5th percentile, median, and 95th percentile of the dN/dS ratio were calculated for *A. montbretiana*/*A. alpina* and *A. thaliana*/*A. lyrata* gene pairs in these gene families (Figs. 4 and 5). The dN/dS values for all pseudogenes compared to their functional counterparts are above the 95^th^ percentile with the exception of kinase 1. This suggests that other than kinase 1, the pseudogenization was not recent or that there has been diversifying selection.

**Fig. 6:**
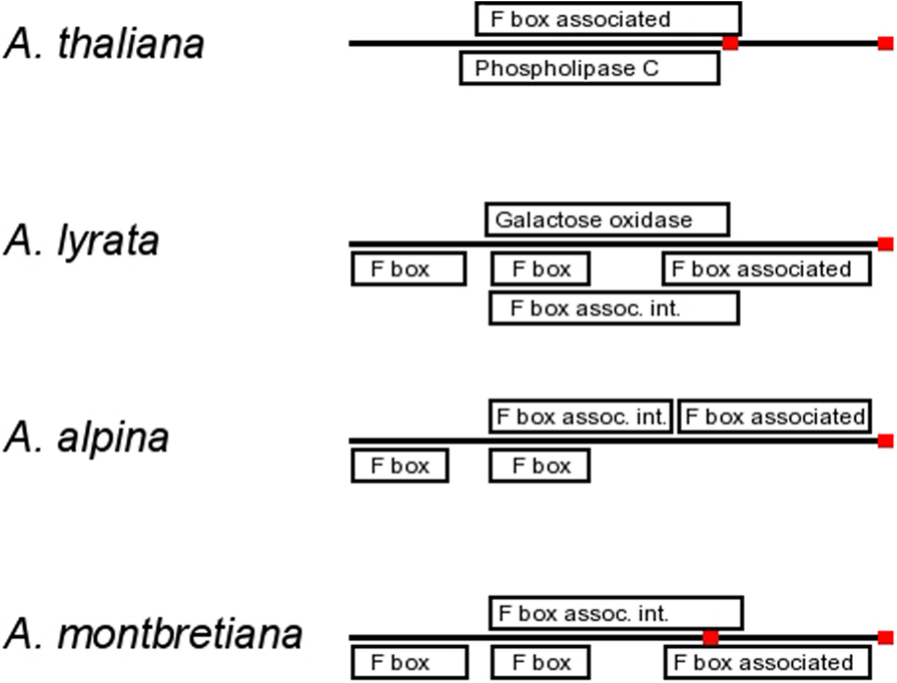
Alignment of domains and premature stop codons in four species for the F-box gene. The thick black line represents the gene, and red squares indicate stop codons. Full names of the IPR protein domains are as follows: F box–IPR001810 (F box domain), Phospholipase C–IPR000909 (Phospholipase C), F box associated–IPR006527 (F box associated), Galactose oxidase–IPR011043 (Galactose oxidase), F box assoc. int.–IPR017451 (F box associated interaction). The figure is not to scale

Of the five families, all could potentially have some impact on annual/perennial transitions, however the F-box protein is the best candidate. It is not found in the *D. hispanica or A. parviflora* perennial species transcriptomes, but many F-box proteins are expressed at low levels [22] and may not be readily recovered in transcriptome data. Additionally the genes may not have been expressed or only expressed at extremely low levels in the leaf tissue that was sampled for RNA. An at least partially homologous gene or pseudogene is seen in syntenic positions in both *A. thaliana* and *A. montbretiana* indicating that we did not miss the true orthologous genes due to sequencing or assembly errors. In *A. thaliana,* the gene is an annotated gene but has gained a premature stop codon and has lost the F-box protein domains while gaining a candidate Phospholipase C domain (Fig. 6). These changes are enough to cause the gene not to be clustered with the other genes in the OrthoMCL algorithm and are also enough to consider the gene “lost” in *A. thaliana* compared to *A. lyrata* for the purpose of this analysis. In *A. montbretiana* there is a pseudogene with an open reading frame that contains F-box domains, but a stop codon breaks both other F-box related domains (Fig. 6). The gain and loss of F-box genes found here is consistent with previous studies finding frequent F-box gene birth/death events in plant lineages [16, 45].

Further support for the F-box genes is in the dN/dS ratios and the function of the genes. The dN/dS ratios suggest that the gene in *A. thaliana* and the pseudogene in *A. montbretiana* have not merely had minor recent changes, therefore changes in the genes began earlier and are possibly important for species differences. The F-box and kinase families are also the only families where pseudogenes with a high dN/dS ratio were found in both pairs of species, indicating that the genes are certainly missing in the annuals and not missed due to incomplete sequencing. Additionally, F-box proteins are involved in signal transduction by regulation of protein stability through ubiquitination, and as previously mentioned the *A. thaliana* gene is potentially involved in vernalization, a process that differs between annuals and perennials [20, 43]. Functional studies of this gene can further determine the role of this gene family in annual perennial transitions.

## Conclusions

We compared four species pairs each containing one annual and one perennial and identified five candidate gene families where the gene is present in the perennial but has been lost or greatly altered in the annual. This suggests that they may be involved in the life history switch from perennial to annual. The five families include an oxidoreductase, a lactoylglutathione lyase glyoxalase i-like gene, a kinase, a zinc finger and a F-box gene. The F-box gene is the best candidate of these five families for future functional studies due to a combination of several findings. Pseudogenes or highly changed yet homologous real genes for the F-box gene could be identified in both annuals for which genomes are available. The F-box family also shows high dN and dS values compared to the median suggesting that the genes were not lost in the annuals not only very recently and therefore may be important in the life history switch. Finally the involvement of F-box proteins in signal transduction is consistent with life history switches.

## Additional files

Additional file 1: Table S1. Information on *D. nemorosa*, *D. hispanica*, *A. parviflora*, *A. verna* and *A. montbretiana* including their accession numbers, source locations and donor. (XLSX 186 kb)
Additional file 2: Table S2. Provides Genbank accession numbers for the genes used in the phylogenetic tree. (XLS 20 kb)
Additional file 3: Table S3. Provides Genbank accession numbers for *A. alpina* genes and Phytozome V9 identifiers for *A. lyrata* genes in the five final families that contain no genes from annuals. (XLS 20 kb)
Additional file 4: A Newick text file containing the phylogenetic tree in Figure 1. (TXT 283 bytes)

## Abbreviations

BBH: Bidirectional best hits
dN: nonsynonymous substitutions per nonsynonymous site
dS: synonymous substitutions per synonymous site
